# Custodians of Information: Patient and Physician Views on Sharing Medical Records in the Acute Care Setting

**DOI:** 10.1080/10410236.2020.1803553

**Published:** 2020-08-19

**Authors:** Zoe Fritz, Frances E. Griffiths, Anne-Marie Slowther

**Affiliations:** a THIS (The Healthcare Improvement Studies) Institute, University of Cambridge; b Warwick Medical School, University of Warwick

## Abstract

In the UK, in the acute in-patient setting, the only information that a patient receives about their medical care is verbal; there is no routine patient access to any part of the medical record. It has been suggested that this should change, so that patients can have real-time access to their notes, but no one has previously explored patient or clinician views on the impact this might have. Semi-structured interviews were conducted with 12 patients and 13 doctors about their experience of information sharing in the context of the acute care setting, and their views on sharing all of the medical records, or a summary note. Interviews were transcribed verbatim, double coded and analyzed using the constant comparative method. Patients were not given written information and did not ask questions even when they wanted to know things. Patients and doctors supported increased sharing of written information, but the purpose of the medical record – and the risks and benefits of sharing it – were disputed. Concerns included disclosing uncertainty, changing what was written, and causing patient anxiety. Benefits included increased transparency. Use of a summary record was welcomed as a way to empower patients, while doctors felt they had a responsibility to curate what information was given and when. A clinical summary for patients would be of benefit to doctors, nurses, patients and their relatives. It should be designed to reflect the needs of all users, and evaluated to consider patient-relevant outcomes and resource implications.

## Introduction

The medical record – also referred to as the patient’s ‘notes’ – is a working document for clinicians. It has several functions including that of a repository for clinicians’ thoughts, a means of inter-professional communication (Lee et al., 2017); a legal document and record of events during a patient’s stay. It includes differential diagnoses, planned investigations, results of investigations and treatments. It does not present areas of clinical certainty or uncertainty in an ordered way. Trained administrative staff are often unable to extract relevant information (Nouraei et al., 2015). Legally, in most countries, the medical record remains the property of the institution in which it is written, but the patient has the right to access it. HealthIT.gov (https://www.healthit.gov/faq/what-are-differences-between-electronic-medical-records-electronic-health-records-and-personal) states that medical records can be in paper or electronic format; in hospitals with electronic medical record systems patients can be given access to selected parts of their record (for example, their blood tests or their clinic letters) through a secure patient portal. A systematic review (Kelly et al., 2018) of the design, use and impact of in-patient portals identified patient interest in these but found little research on their use or impact (Kelly et al., 2017; O’Leary et al., 2016; Pell et al., 2015). A systematic review of patient access to their medical records in the acute setting (D’Costa et al., 2020) identified 12 empirical papers none of which investigated patient and physician perspectives of access to real time complete medical records. Citizens in countries such as Denmark, Estonia and Australia (Nohr et al., 2017) and Sweden (Armstrong, 2017) have access to their health data. The Danish health portal allows patients to see their notes and results in real time via Sundhed.dk (www.sundhed.dk) but no studies have been published which evaluate its use or impact.

In the UK, in the acute in-patient setting, the only information that a patient receives about their medical care while they are an in-patent is verbal; although legislation as explained in the House of Commons Library (https://www.nhsconfed.org/resources/2015/10/legislation-and-guidance-relating-to-medical-records-explained-by-house-of-commons-library) states that patients should soon be able to have real-time access to their notes, no one has previously explored/investigated patient or clinician views on the impact this might have (D’Costa et al., 2020).

Between 2016 and 2019 we conducted a qualitative research study exploring clinician-patient communication and patient trust in the acute medical setting. Within this wider enquiry, we specifically explored patients’ and doctors’ views on how real-time patient access to medical records (paper or digital) might change experience or practice.

## Methods

### Study setting

Participants were recruited from November 2016 to January 2018 from two UK hospitals: a large university hospital with patients from urban and rural areas, and a middle-sized urban hospital serving an ethnically diverse population. All emergency care in the UK is provided by the National Health Service.

### Eligibility, sampling and recruitment

Patients admitted with an acute medical problem to the medical admissions unit, who were over 18, had the capacity to consent, and were able to read an information sheet in English were eligible. Samples of up to seven consecutive patients were approached by the medical team or by a research nurse about the study, allowing time for data collection between batches. Patients were invited to sign an expression of interest form, including consent for the researcher to contact them. A participant information sheet was posted to the patient and the researcher (ZF) telephoned to answer questions about the study and arrange a time for interview.

Doctors working in acute medical wards were recruited via e-mail including an information sheet and contact details of the researcher to respond if they were interested in participating. Responding doctors were contacted by the researcher to discuss the study and arrange a time for an interview.

Interviews were conducted at a time and place convenient for the participant, usually a private room in the hospital or the patient’s home. Consent was taken immediately before the interview. The researcher, ZF, is a consultant doctor in acute medicine. Patient participants were told that she was a doctor conducting research who did not know their medical history. Doctor participants were informed of her specialty background.

### Data collection

Interviews were semi-structured: the topic guide (see Appendix A) was informed by a literature review of medical record sharing and conceptual analysis of trust and information sharing (Cox & Fritz, 2016; Fritz & Holton, 2019). Patients were asked to talk about their recent admission and doctors were asked to reflect on recent cases and their wider experience of seeing patients. Interviews explored participants’ views on what factors helped build or breakdown trust, what and how information should be shared, and whether patients should have real-time access to their medical record (i.e be able to see all of the contents of their medical record while they are an inpatient, as things are written, and as results come through). Toward the end of each interview the interviewer asked participants about producing a summary record for the patient. In this paper, we will present the findings related to information sharing.

Interviews continued until data saturation was reached. Field notes were made on points stressed by participants, and emerging themes identified by the interviewer throughout the process of data collection, with interviews being iteratively developed to further explore these themes. Interviews were recorded and transcribed verbatim. Transcriptions were uploaded into the NVivo analysis software version 11.4.3.

### Data analysis

The initial coding framework was based on the interview guide; new codes were iteratively developed (Charmaz, 2006). A-MS and FG independently read 20% of the data and met with ZF to discuss the data and identify new codes, and relationships between codes and existing evidence and theory. All data were coded and extracted. Extracted data were compared across participants.

### Ethical approvals and reporting criteria

Approvals were gained from East of England Cambridgeshire and Hertfordshire Regional Ethics Committee (REC), the Health Research Authority, and Research and Development Departments of participating institutions. In reporting this study, we have applied the 32-item Consolidated criteria for Reporting Qualitative research (COREQ) checklist for in-depth interviews (see Appendix B).

## Results

### Study participants’ characteristics and narratives

Of the 46 patients approached, 34 signed an expression of interest form and 12 were interviewed (8 from site A; 4 from site B). Patients reported a range of conditions including cellulitis, pulmonary embolism, falls, pneumonia and chest pain. Of the 50 doctors who were contacted, 13 were interviewed (8 from site A; 5 from site B). Doctors came from a range of cultural backgrounds with between 2 and 30  years’ experience working as a consultant.

#### Interviews ranged from 20 to 53 minutes

We first describe participants’ experiences of the acute care setting in relation to information sharing. We then present participants’ views on potential changes to patient access to clinical information, including access to the whole medical record, or a summary record.

### Information sharing in the acute medical setting

Doctors recognized the importance of sharing information, of being open, and of not hiding anything:

I quite openly say I just want you to know that I am not hiding anything any information from you, as soon as I have information I will give it to you and what I have discussed with you thus far is all I know, there is nothing else that I’m keeping from you. (Dr B7)

This openness was also valued by patients:

… whatever range of doctors they are, they must show an attitude of friendliness and openness. The openness being you can ask them anything. (P6)

Nevertheless, five patients complained about not being told enough about their condition, while others had not understood what they were told. If they did understand what was said, remembering it was a challenge:

Beautifully explained, very clear, I could absorb it all and listen and understand and everything and then by the time you [her husband] got there it was sort of half and half. (P4)

Patient participants recognized that the busy ward environment and feeling ill or anxious made retention of information difficult:

… there’s a lot of other beds there as well, you’re feeling really ill, you haven’t got your family around you, and you just forget. (P8)

Doctor participants were aware that patients did not always – or even often – remember what they had been told:

It’s not uncommon [for patients] to have no recollection. (Dr A1)

Z: So in your experience, how much of what you’ve told them on the post take round do you think they take in?

D: maybe half … In some patients more, in others less.

Z: And how much do you think they remember to be able to pass on to relatives?

D: Based on the number of questions I have had from relatives subsequently: very little. (Dr B2)

No patient participant reported seeing their medical record while an inpatient, and no doctor participant reported showing the medical record to a patient, although they did sometimes describe sharing specific results.

In fact, despite valuing openness, many doctors perceived themselves as guardians of a patient’s clinical information: not passing all information to patients immediately but sifting through for what was relevant at that time:

… sometimes too much information at the wrong time with the wrong context can actually cause undue stress. So it’s a judgement call I think on the part of the clinician. It’s a fine balance between withholding information and giving information that is relevant and important at that time. (Dr B7)

### Sharing the medical record

The concern about causing stress or anxiety influenced both patients’ and doctors’ views about sharing the medical record. Doctor participants wondered if they would be inhibited from writing things down as they currently do, knowing patients would see it in real time:

Well currently I’m in the habit of writing down a range of possibilities, they are about what I think might be wrong with the patient without necessarily discussing each possibility in detail with the patient. I often write down things that might be quite distant possibilities, but are still on the list – such as cancer – and patients may not appreciate that kind of hierarchy of probability. They often hear that word and go into a tail spin. (Dr2)

Some, some people would be able to process what they see and deal with it and some people it would frighten them what is written down. And that would maybe make their symptoms worse, that would make them more ill, that would stress them, they could become really stressed about reading it. No! I, wouldn’t, no … (P8)

Several doctor participants talked about the possibility of inducing ‘unnecessary anxiety’ by communicating diagnostic uncertainty or through disclosure of slightly abnormal blood tests or incidental findings on CT tests which had no therapeutic significance. While doctors reported that they are open with patients, they also regularly choose not to pass on certain information to patients, using their clinical judgment that these results are unimportant:

… So everybody gets a liver function test in the emergency department. Quite often they are mildly deranged due to general un-wellness, infection or medications and if they’re very mildly deranged I tend not to even mention it. But if I had to explain to every patient why their bilirubin was 23 as opposed to 20 … You know I think it would get them unnecessarily worried over things that I didn’t even think were relevant. (Dr7)

Doctors were concerned that sharing these incidental findings with patients might not only cause anxiety, but take extra time and lead to further unnecessary investigations and use of resources in addition to distracting from the main problem.

So to be able to weigh up a CT scan report and say ‘actually that’s minor I don’t really need to worry about that’ and ‘that’s really important I need to worry about that’. That is clinical judgement to my mind. So that is not something that’s easily transferable on a piece of paper and that’s the bit that’s going to take time from the clinician’s part to have to go through. (Dr B7)

… the worry for me is that a patient will see all those yellow flags and get focussed on each individual one … and in trying to explain it [slightly abnormal results] to them it will either take a lot of time or even worse it will then prompt unnecessary investigations that will lead to [revealing insignificant] abnormalities etc. and so take us down that diagnostic odyssey that we don’t want to take. (Dr6)

The role of the medical record as a tool for communicating to other health professionals, not only *diagnostic* uncertainty, but uncertainty about whether there was an ‘organic’ or physical diagnosis for symptoms (as opposed to a psycho-social cause) was raised by several doctors as a reason not to share medical records with patients.

I think it would make it very difficult particularly for those patients who are already a management problem, patients who have somatic, psycho-somatic illnesses, sort of somatisation, [or] manipulative behaviour … you do need a way of communicating with your colleagues … that you are suspicious that this is not a straightforward interaction … (Dr6)

Some doctors could see possible benefits from being more open about somatization disorders, in order to help patients gain insight into their condition and access appropriate therapies:

… at some point that person needs to know that they don’t have true pathological epilepsy and … I do spend quite a lot of time … gently explaining to people that, that I think they’re fine. (Dr7)

### Suggested alternatives or additions to sharing the whole medical record with the patient

Several unprompted suggestions were made to improve communication between doctors and their patients and relatives. One doctor and one patient participant proposed that patients should be able to contribute to their medical records, providing a history as they would at a dental surgery (using a tick sheet). One doctor suggested that *some*, rather than *all* of the notes could be shared – so that information was easily digestible, and not overwhelming.

… a brief summary of possibly differentials and a plan of investigations and treatment that you were doing at that point, something simple like that. (Dr A3)

### Producing and sharing a summary record for patients

When the idea of a summary record was raised both doctor and patient participants were positive about its potential to improve patient care.

#### What to include in a summary record

Suggestions included: the most likely diagnosis, other possible causes of illness (differential diagnosis), treatments started and investigations planned. This is consistent with what doctors said during interviews about what they tell patients when they see them after their emergency admission. One doctor suggested that a summary record should include what the doctor thought the patient had come in with (the so-called “presenting complaint”) so the patient could check this.

I guess you would at that stage you would want to put a brief summary of what you think they’ve come in with so that they were happy that you had got the main points of their complaint. (Dr5)

This would prevent doctors getting caught up in problems they have identified from their investigations, and forgetting the initial presenting complaint of the patient.

There was disagreement about including the predicted length of stay. One doctor suggested the summary record should include a list of what needs to happen before the patient goes home and this proposition was received positively by both patient and other doctor participants.

#### Potential benefits of a summary record

Patients thought having a summary record would help them think in their own time about what questions they want to ask the doctor and that it would also help them communicate with their relatives about what was happening to them:

I can peruse it … it would have given me a chance to ask the question, analyse it in my own time and [the] reasoning behind it … ask for more information if you don’t understand it, that’s the quite important one. (P6)

I think sometimes it’s very helpful to patients who then have relatives come in and go, ‘why are you in?’ ‘Oh I don’t know’. ‘What’s the plan?’ ‘Oh I’m not really sure.’ Who [patients] may have taken it in perhaps, at the time of getting info, but then maybe don’t take it in long-term or don’t remember it very well. I think it could potentially be useful for that kind of thing; reassuring for other family members. (P1)

Doctors suggested that the summary record could be used as a prompt to encourage patients to document their questions;

If patients are going to be given information, they should also be given a pencil and space to ask questions. (P3)

One patient suggested doctors could explain to patients that they were writing down a version of what they had said, and offer to give a copy to their relatives to help keep them in the picture:

… ‘What I’ve done is I’ve written this down again Mrs <Anonymous> and I’m going to give it to your son’ … I think that would be helpful to a lot of people. (P7)

#### Challenges of producing and sharing a patient-facing summary note

Many participants – both doctors and patients – were concerned about the resource implications of preparing the summary note and giving it to the patient. Most doctor participants said that while medical records were on paper, preparing and distributing summary notes would be very difficult; if it was introduced in hospitals using an electronic medical record, it was thought that the extra time taken would be reduced and was considered by most doctor participants to more acceptable.

… well I mean it would be perfect because the post take plan is often very simple and non-controversial anyway … it would be very simple just to print … perhaps it would add a few minute to a round per patient to produce a lay person’s version. (Dr B6)

Both doctors and patients recognized that a ‘one-size-fits-all’ approach would not work: for some patients access to all information would cause anxiety, while others would be reassured that nothing was being kept from them.

P: I don’t think it’s something that you can make blanket rules for.

Z: No.

P: People tend to ask for information I think in one way or another. (P1)

So if you’re a patient that wants to know absolutely everything and not knowing everything is going to cause them more stress, then you have to accordingly adjust what information you’re giving. [then] you have somebody that says ‘doctor I don’t really want to know anything, You know let me know what’s important’. So it’s very situational, I don’t think you can make a blanket rule. (Dr B7)

Doctors identified that many factors contribute to variation in patient expectation, particularly in the context of discussing uncertainty in diagnosis:

… it partly depends on a lot of factors I think; the patients’ age, their education level, their socioeconomic status … [those] from a background where they’re not very privileged they’re more likely to take it at face value, and say ‘yeah whatever the doctor says is correct’. (Dr B4)

One participant suggested that this variability in patients’ responses may lead to inequity with some patients receiving more information and more attention from their doctors than others:

… widen the gap of care between those informed, educated individuals and those more passive, trusting individuals. One may pour over their notes, occupy a lot of time or resources whereas others will just not look at them. (Dr B1)

Care would need to be taken to mitigate against this – to ensure that those less confident patients would also be empowered to ask questions from the written summary.

Doctors also expressed concerns that there might be legal implications to providing a written summary, which might make them hesitant to do it:

I think we feel like written word is more legal than the spoken … I think people worry about what they write down in the absolute 100% accuracy that it has to have if it’s going to go to a patient. (Dr B2)

Doctors emphasized that any summary should be personalized to reflect the needs and understanding of each patient, and that it would need to include an explanation that the situation, and therefore the information, in an acute care setting is likely to change often quite quickly.

Understanding their level of understanding – so educational levels etc. – is really important … background is very important, how they want that information to be given is really important. (Dr B7)

It was also emphasized that any summary should come with an explanation that things can change:

I think sometimes when you have written information, if you then deviate from the plan people can find that very stressful or anxiety-causing or provoking. (P1)

… the only caveat is obviously in an acute setting things change very rapidly. So what’s true at that moment in time may change quite rapidly. (Dr B7)

Despite all of these potential barriers, it was noted by one doctor that clinicians now write outpatient letters directed to the patient, in lay terms, despite similar initial concerns expressed by professionals.

### Strengths and limitations

This study was conducted in two hospitals in England, and although there was a wide range of age and ethnicity in doctors in the sample, the patient participants were predominantly White British; care should therefore be taken not to extrapolate these results to other cultures or other health care settings. As a practicing doctor, ZF was aware of many of the common practices that were referred to and medical language used, particularly by doctors. This was both a strength and weakness in conducting the interviews; she was able to understand the context and started from a level of trust with the doctor participants, but they may have not been explicit about some of their reasoning, assuming that she would already understand.

Participants may have exhibited social desirability bias in wanting wanted to please a doctor-researcher, and so their positive reaction (for example, to the summary record proposal) needs to be interpreted in this context.

## Discussion

This paper – and the questionnaire study from the same programme of work (Fritz et al., 2019) – is the first to present patients’ and doctors’ views on sharing the medical record in the acute care setting.

Key findings were that patients were not given written information and did not ask questions even when they wanted to know things. Both patients and doctors saw openness as essential to the patient-doctor relationship; overall, patients and doctors support increased sharing of written information, but the purpose of the medical record – and the risks and benefits of sharing it – were disputed. Concerns about the unintended consequences of sharing the medical record included disclosing uncertainty, changing what was written, and causing patient anxiety. Doctor participants recognized it might force them to be more transparent about currently hidden matters such as differential diagnoses and suspicions about somatization. Use of a summary record was welcomed as a way to empower patients. Doctors also valued the ability to maintain responsibility for curating what information was given and when, in order to minimize patient anxiety and maximize understanding. The possibility that such a change might add to inequity of health care utilization would need to be mitigated against.

### The role of the medical record

Questions about sharing the medical record revealed conflict among our participants about what its role was. Although the importance of the medical record is recognized (Royal College of Physicians, 2015), and guidance is supplied by the Professional Record Standards Body on how to do this to a particular standard (www.theprsb.org), there is very little in the medical literature about its function. There is more in the legal literature. In a medical law review article by Heywood, he argues that:

The main aim of the notes is to chart a comprehensive history, which can then be read by other medical colleagues; they must appear in an accessible and decipherable format in order to avoid the problem of a GP not adequately considering what has gone before when attempting to reach an accurate diagnosis. (Heywood, 2019)

While he is talking primarily about GP notes, this description applies to all medical records. Medical records now take many forms: the paper record still exists in many places, while in others full electonric or digital records, allow information to be ordered and accessed differently. Digital records also obviate concern about poor handwriting, and enable sections of the record to be easily shared with patients and/or their relatives, as the OpenNotes project has demonstrated (Delbanco et al., 2012;  Walker et al., 2011) The purpose and ownership of a medical record has debated in the courts. In R. v Mid Glamorgan Family Health Services Authority and Anr. [1993] P.I.Q.R. P426. Mr Justice Popplewell, sitting in the High Court, commented that

… the opinion of the doctor is wholly the property of the doctor. It does not seem to me that the fact that the patient provides the original information entitles him subject to exceptions, to see the conclusions of the doctors based on that information.

Furthermore, in the Court of Appeal Lord Justice Evans stated that the record is created to ‘provide part of the medical history of the patient, for the benefit of the same doctor or his successors in the future’. More recently, the Data Protection Act 2018 (and, prior to this, the Access to health Records Act 1990) has enshrined in law the right of the patient to access their medical records and ensure that the information contained in them is correct – but the Act does not specify the purpose of the record.

### Open notes as a way to empower patients and increase questioning

Current lack of access to their medical record means that patients are ill-equipped to ask questions of their doctor, or become actively engaged in their care. Several studies have suggested that the majority of patients have poor recall of what was said to them by doctors (Gignon et al., 2014; McCarthy et al., 2012), and this was reiterated in our research.

The patients interviewed for this study revealed they had not asked questions despite wanting answers; this has been termed ‘white coat silence’ (Judson et al., 2013), and attempts have been made to address it in quality and safety research (Osborne, 2008).

It is possible that having real-time access to records would improve patient engagement and questioning. The change was made in outpatient documentation two decades ago despite initial concerns expressed by professionals (White et al., 2004). In the US, access to outpatient medical records has been evaluated and is now commonplace (Delbanco et al., 2012).

Giving patients access to their medical notes would mean that doctors would have to be more open about currently undisclosed matters such as differential diagnoses and suspicions about somatization. This might in turn allow exploration and resolution of unspoken fears. Patient access to their records may also improve patient safety: patients could pick up on prescribing errors, or alert doctors to delayed test results (Callen et al., 2015).

### Changing access: Changing content?

Doctors’ concerns about sharing the whole medical record were in part based on a fear of losing their discretionary judgment about *what* information to give and *when* in order to minimize psychological harm to each patient. Giving lots of information at once has been shown to be overwhelming (Ubel et al., 2017). While withholding information from patients may be perceived as paternalistic behavior, it can also be seen as part of a clinician’s responsibility, both to the individual patient and to the system (Specker Sullivan, 2016).

Research is needed to investigate what changes occur – in what is written, in the patient–doctor interaction and in patient experience – when written medical information is routinely shared. So far, the evidence has been limited: early adopters of ‘patient portals’ – a patient-tailored view of part of the electronic medical record – have published papers on the development and implementation of their approaches, but have not evaluated the impact of such portals (Grossman et al., 2017; Wilcox et al., 2010). A Randomized-Controlled Trial of a patient portal for cardiology patients is underway (Masterson Creber et al., 2016): the primary outcome measure is patient engagement. A recent review of the literature did not reveal any studies investigating the impact of sharing medical records on patient empowerment, changes in documentation, training of junior doctors, or interactions between patients and the multi-disciplinary team, including the resource implications of increased patient engagement and questioning (D’Costa et al., 2020).

Further research is required to investigate the impact of sharing patient records on these outcomes along with assessing changes in medication adherence and medical errors (Tennstedt, 2000).

Importantly, interventions may unintentionally increase inequalities by disproportionately benefiting more advantaged groups, (Lorenc et al., 2013) work should be done to evaluate whether equipping patients with more medical information would introduce inequities between well and less well-educated patients or alternatively empower less confident patients to ask questions.

### Conclusion

The medical record as it currently exists has developed reactively – in most part in response to doctors’ needs. A medical record, which will be of real benefit – to doctors, nurses, patients, and relatives – needs to be designed with an understanding of the needs of all the users. This paper provides insights to inform further research and policy development. It may be that rather than sharing what we already have, we should redesign (and rename?) the patient clinical record, or create a patient clinical summary and evaluate its impact on all users: patients and relatives, doctors and nurses.

## Appendix A. Topic guide

**Semi Structured Interview Guides**

*For Doctors*

Please tell me about your career as clinician until you reached this acute setting.

Tell me something of your most recent day of clinical duties – patients you saw, where you were working and so on to give me an idea of the general day.

(any topics of interest from this will then be explored further, for example, if the doctor mentions interactions with patients, patients they found particularly challenging, details of their ‘ post take round’ – how many patients, how long with each, whether they will see them again)

How would you describe the role of trust in the Doctor- Patient relationship?

Is there anything you do to encourage your patients to trust you?

What kind of thing do you think might make a patient distrustful?

Do you ever tell your patients personal things about you/ask them questions that are not relevant to their medical condition?

(If so) What do you think the purpose of this is?

Can you tell me about a patient you saw recently who you found it hard to gain trust from?

Why do you think it was difficult?

What do think it that made it difficult?

What might have made it easier?

Can you tell me about a patient you saw recently who you had a good relationship with?

Why do think it was good?

Do you think if the patient had access to his/her medical records it would have changed the interaction?

If so how?

Do you tell your patients about treatment decisions?

Can you give me an example of this?

Do you ever show your patients what you have written down?

Can you give me an example of this?

Can you tell me why you showed them what you had written?

When might you not do this?

Why is that?

Can you think of an example of a patient who asked you questions about their diagnosis or treatment decisions?

How did you feel?

Do you think it changed the way you interacted with them?

Do you think it changed the information you gave them?

Do you think it changed your management of the patient?

If you were a patient, would you want to see your medical records?

If you were a patient, are there any other things you would want access to?

(if so) Why?

Do you think having access to the medical records would have changed any of the interactions you mentioned above? ? If so how?

In general what do you think of the idea of sharing medical notes with patients?

Do you think there are some things which should not be shared with a patient?

If written information was to be shared with patients, what do you think it should include, and when should it be shared?

*For Patients*

Please tell me about yourself (prompts: who do you live with, work/past work, daily activities etc).

Can you tell me something about your recent admission into hospital from the first moment you realized you might need to go in to hospital.?

**(any specific topics of interest from this will then be explored further)**

What kinds of information were you given?

Were there times when you wished you were given more information?

Were there times when you felt you were given too much information?

Did you feel you had the opportunity to ask questions?

Did you ask questions?

Did you feel you could trust your doctor(s)?

What do you think led to that?

Do you think you are a naturally trusting person, or do people need to earn your trust?

Do you think that being given more information would have changed how much trust you felt?

Would you like to have access to your medical records?

If you were creating the health system afresh, would you change anything about the way we share medical notes/information?

Would you like to have access to all of your medical records?

Why?

Can you think of any problems there might be with having access to all of the records?

Can you think of any benefits there might be?

If written information was to be shared with patients routinely, what do you think it should include, and when should it be shared?
